# Foreword: Focus on complex metallic phases

**DOI:** 10.1088/1468-6996/15/5/050302

**Published:** 2014-09-08

**Authors:** Dan Shechtman

**Affiliations:** >Department of Materials Science, Technion-Israel Institute of Technology, Israel

## Abstract

I am glad to learn that a focus issue, Complex Metallic Phases, will be published in *Science and Technology of Advanced Materials* (STAM). The articles in this special issue contain original and review papers on fundamentals and applications contributed by experts in the field. The topics cover various approaches to practical applications including catalysts, photonics, high specific strength materials, thermal storage materials and thermal rectification for quasicrystals, as well as related complex compounds. It is exciting to see the great potential of these complex phases, and I look forward to seeing their industrial applications in the foreseeable future.

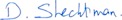

Professor Dan Shechtman

Recepient of the Nobel Prize in Chemistry 2011 for his discovery of ‘quasi-crystals’ in 1984.

